# Audit: lithium monitoring for psychiatric inpatients and community patients during the initiation phase

**DOI:** 10.1192/bjo.2021.227

**Published:** 2021-06-18

**Authors:** Damodar Chari, Mohammed Abbas, Kajal Dhesi

**Affiliations:** Leicestershire Partnership Trust

## Abstract

**Aims:**

Measure compliance with standards requiring baseline work up before Lithium therapy is commenced and subsequent Lithium level monitoring during the initiation phase

**Method:**

All inpatients and outpatients who were started on Lithium between 2018 and 2019 within the Leicestershire Partnership NHS trust. Case notes were of patients 128 were retrieved from the electronic system and an audit proforma was completed to ascertain adherence to auditing standards as per BNF and trust guidelines. Parameters monitored were full blood count (FBC), renal functions test including serum electrolytes, thyroid function test, and BMI before commencing Lithium, and serum Lithium periodically after. ECG was needed for those patients with cardiovascular illness. Data were systematically compiled and analyzed descriptively using Microsoft Excel

**Result:**

A total of 128 patients were included in the study. 111 (86.71%) had FBC, 118 (92.19%) had renal function test and electrolytes, 114 (89.06%) had thyroid function test while 99 (77.34%) had their BMI/weight recorded before initiating Lithium. 26 out of 36 patients with cardiovascular disorder had their ECG recorded. After Lithium was commenced, 108 (84.37%) had serum Lithium tested a week later, while only 89 (69.53%) had lithium monitored weekly. Trust guidelines recommend weekly monitoring for up to 4 weeks after a stable dose was reached. This was monitored in only 16 out of 128 patients.

**Conclusion:**

Most of the patients had blood test done before being commenced on Lithium. However it was observed that serum Lithium was not adequately monitored at regular intervals after dose escalations. These finding indicate that there has to be greater awareness of the trust and BNF guidelines with regards to Lithium monitoring.

